# Axillary Aneurism Successfully Treated by Tying the Subclavian Artery

**Published:** 1831

**Authors:** 


### Axillary Aneurism successfully treated by tying the Subclavian Artery. By Mr. Crossing.*

This is the second case of the kind related in the last volume of the Medico-Chirurgical Transactions. We shall mention the particulars very briefly; for, thanks to the progress of modern surgery, such cases are now "plenty as blackberries."

*Case.* A stout muscular man, æt. 46, was admitted into the Parochial Infirmary at Devonport, June 10th, 1830, with the following affection. There is a diffused pulsating tumour, situated immediately under, and closely in contact with, the right clavicle; extending to the cartilage of the fifth rib, stretching into the axilla, and over the point of the shoulder. It has a very tense, elastic feel, and the pulsation is generally rather obscure, but at other times is so distinct as to be seen at a considerable distance from the patient. The tumour is not compressible, but the pulsation can always be stopped by pressing on the artery above the clavicle. The arm from the shoulder to the extremities of the fingers is swollen to an enormous size; is benumbed, and has lost all power of motion. The pulsation at the wrist cannot be felt, and the arm is kept nearly at a right angle in consequence of the magnitude of the swelling in the axilla; the pectoral muscle and integuments covering it being stretched to the greatest extent. He is always in pain, and at times to a most agonizing degree; is unable to lie back in the bed, but is continually in a sitting position, with the arm supported on a pillow, and the body bent forward. His countenance is marked with great distress.

Thirteen weeks previous to his admission he was attacked, after exposure to cold as a fisherman, with a little tumefaction above the right elbow-joint, and pains supposed to be rheumatic. A

* Med. Chir. Trans. Vol. XVI. Part II.

month after this he discovered a tumour, the size of a walnut, midway between the clavicle and tendon of the pectoral muscle, which in a fortnight attained its greatest size. Mr. Crossing bled the patient occasionally and put him on a low diet. On the 23d of June, having obtained the sanction of Dr. Thomas, and Messrs. Lower, Williams, and Baldy in consultation, he proceeded to the operation.

"The patient was seated in an arm chair, the head directed to the left side, and supported by Mr. Buchan, one of the gentlemen who kindly gave me their assistance. The integuments over the clavicle being stretched upon the chest, I commenced my incision near the sternal attachment of the mastoid muscle, and cut freely on the bone for about three inches and a half; thus dividing at once the integuments and platysma myoides. The parts being now allowed to retract, left the lower margin of the incision half an inch above, and running nearly parallel with, the clavicle; and exposed the jugular vein to a considerable extent, which was easily drawn aside, and kept out of the way, with a blunt hook. The cervical fascia was next carefully divided from the clavicular edge of the sterno-cleido mastoideus to near the extremity of the wound, which brought into view the omo-hyoideus. This muscle instead of forming a triangular space, as it does in most instances, with the scalenus anticus and clavicle, ran in a line with, and just above, that bone. Finding this rather unusual course of the omo-hyoideus an impediment, I passed a director under, and divided it. The knife was now laid aside, and the remaining part of the operation finished with the fingers, and a common director. Some loose cellular membrane, and a large fatty gland being removed, the artery was found immediately below this substance, and three considerable branches of nerves passing over the vessel, and in close contact with it. These were separated, and the ligature passed under it.

This part of the operation I found the most difficult. It required considerable force, and care to pass the point of the needle under without injuring the vessel, or separating it to a greater extent than is desirable, from its surrounding connexions; but using Weiss's ingenious instrument, both these injurious effects were obviated, and the ligature brought up from the eye of the needle with great ease. I then raised the artery, and finding the pulsation of the tumour cease and nothing besides included, tied it with a double knot just before it passes over the first rib. One end of the ligature was cut off close to the artery, the other left hanging from the wound, the edges of which were now brought together, and secured with one suture and adhesive straps. During the operation, which was borne with great fortitude, two arteries were divided, and immediately taken up; and the patient on the whole did not lose more than two drachms of blood. From the time of leaving his bed to his return, a period of twenty-five minutes elapsed."

All went on well after the operation, and on the 2d of July the wound was healed, with the exception of a small space where the ligatures were. On the 6th one of the ligatures on a small artery separated, and in the evening slight hæmorrhage took place. He was bled, and sore throat with fever supervening, the bleeding was required twice more. From this time nothing of consequence occurred. The ligature on the subclavian did not come away till the eighty-fifth day. In December, 1830, when the last report is given, the man's general health was as good as it could be, the circulation throughout was perfect, and nothing of the tumour was left but a little thickening in the aneurismal sac, along the edge of the pectoral muscle. We think that the case altogether is highly creditable to Mr. Crossing.

